# A new red cell index and portable RBC analyzer for screening of iron deficiency and Thalassemia minor in a Chinese population

**DOI:** 10.1038/s41598-017-11144-w

**Published:** 2017-09-05

**Authors:** Lieshu Tong, Josef Kauer, Sebastian Wachsmann-Hogiu, Kaiqin Chu, Hu Dou, Zachary J. Smith

**Affiliations:** 1 University of Science and Technology of China, Department of Precision Machinery and Precision Instrumentation, Hefei, Anhui China; 2 Beuth Hochschule für Technik Berlin, Berlin, Germany; 3 Center for Biophotonics Science and Technology, University of California, Davis, Sacramento, CA USA; 4 University of California, Davis, Department of Pathology & Laboratory Medicine, Sacramento, CA USA; 5 McGill University, Department of Bioengineering, Montreal Quebec, Canada; 6 Department of Clinical Laboratory, Ministry of Education Key Laboratory of Child Development and Disorders; Key Laboratory of Pediatrics in Chongqing; Chongqing International Science and Technology Cooperation Center for Child Development and Disorders, Children’s Hospital of Chongqing Medical University, Chongqing, China

## Abstract

Anemia is a widespread public health problem with 1/4 ~1/3 of the world’s population being affected. In Southeast Asia, Thalassemia trait (TT) and iron deficiency anemia (IDA) are the two most common anemia types and can have a serious impact on quality of life. IDA patients can be treated with iron supplementation, yet TT patients have diminished capacity to process iron. Therefore, distinguishing between types of anemia is essential for effective diagnosis and treatment. Here, we present two advances towards low-cost screening for anemia. First: a new red-cell-based index, Joint Indicator A, to discriminate between IDA, TT, and healthy children in a Chinese population. We collected retrospective data from 384 Chinese children and used discriminant function analysis to determine the best analytic function to separate healthy and diseased groups, achieving 94% sensitivity and 90% specificity, significantly higher than reported indices. This result is achieved using only three red cell parameters: mean cell volume (MCV), red cell distribution width (RDW) and mean cell hemoglobin concentration (MCHC). Our second advance: the development of a low cost, portable red cell analyzer to measure these parameters. Taken together, these two results may help pave the way for widespread screening for nutritional and genetic anemias.

## Introduction

IDA is caused by the deficiency of iron, which is used in the manufacture of hemoglobin. The reduction of heme synthesis leads to the characteristic microcytic, hypochromic anemia. It has a high incidence among children and among women of childbearing age^1, 2^. According to the World Health Organization (WHO), the worldwide incidence of adult females is ~20%, ~40% for pregnant women, ~47% for children under 5 years of age, and ~10% for adult males^3^. Totally, about 2 billion people around the world have anemia, of which more than 90% are IDA. Health problems caused by IDA include decreased physical strength (especially endurance), distraction, thinking and memory loss, and reduced learning efficiency^4, 5^. It may also reduce the body’s immunity to various infections^6^. Pregnant women with anemia are at increased risk for pregnancy complications, and anemic infants have potentially reduced physical and mental development^7^. IDA is easily preventable and can be treated simply through iron supplementation. However, broad-spectrum iron supplementation is problematic due to the negative impact of iron on those with other forms of anemia, as highlighted by the classic “Pemba” study^8^. Therefore, IDA should be separated from other genetic anemias prior to treatment.

Among genetic anemias, Thalassemia minor, or Thalassemia trait (TT) is one of the most common types. It is characterized by the absence or decreased accumulation of one of the globin subunits^9^. The most common forms are α-thalassemia and β-thalassemia, which affect the synthesis of α- and β-globin subunits respectively. It can result in chronic hemolytic anemia. Inherited hemoglobin (Hb) disorders are the most common inherited blood disorder in the world^10^. TT is the most common genetic anemia present in China, and its prevalence especially in the Southeastern portion of the country is widespread^11^, with estimates that as much as ~10% of the population in SE China carry Thalassemia trait^12^. Although TT cannot yet be cured, diagnosis is helpful for genetic counseling and routine monitoring. As mentioned above, any widespread anemia screening should distinguish IDA and TT prior to treatment, particularly given TT patients’ lowered ability to process iron. However, both IDA and TT are endemic primarily in lower resource settings and remote areas that often have poor medical infrastructure. In addition, anemia often has mild, vague symptoms such as fatigue. Due to the economic consequences of leaving work to seek medical care, those with mild anemia may not want to see a doctor until their anemia causes severe consequences. Yet current tests are restricted to a central laboratory where the whole detection process is very complex and must be administered by costly, highly-trained personnel^13^.

To help resolve this conflict and increase access to care, a new screening paradigm is needed, where a simple to administer, sensitive screening test can be used to identify anemia, and separate it into IDA vs. TT. This will minimize use of scarce resources while at the same time maximize the percentage of the population screened for disease. Hennig *et al*. recently reported an elegant and promising experimental approach for non-invasive determination of IDA using zinc protoporphyrin fluorescence^14^. However, because it cannot identify TT, it does not meet the screening needs of areas where TT is endemic. Since the late 1970s there have been attempts to use automated red cell analyzers to discriminate IDA and TT. Several previous studies have confirmed the ability for red cell parameters to discriminate between iron deficiency anemia and thalassemia minor^15^, and even between thalassemia trait from combined sickle-cell thalassemia^16^. However, the performance of these metrics has varied in the literature, with some authors being quite critical of their prospects for use in widespread screening^17–19^. Poor performance in some studies is likely due at least in part to the differences in specific underlying mutations leading to Thalassemia in different ethnic subgroups^20^. A further problem is that previous metrics are composed of simple arithmetic combinations of the red cell parameters in ways that lack a strong statistical basis, possibly limiting their performance and robustness. Furthermore, these diagnostic indices still require an automated blood analyzer, which is likely not present in low-resource settings. Thus, despite their reasonable diagnostic ability, their use in actual clinical practice has been limited.

Currently, several groups are currently exploring the use of low-cost instrumentation based on mobile phones and other consumer devices blood measurements in low-resource settings^21–24^. However, to date none have addressed anemia screening. Several researchers have demonstrated in the past few years the ability of quantitative phase imaging (QPI) and a related technique, Fourier Transform Light Scattering^25^ (where QPM images are numerically propagated to the Fourier plane, allowing extraction of scattered intensity versus angle for individual cells), to accurately report the size, volume, hemoglobin concentration, and other parameters of red blood cells at the single-cell level^26–28^. While these methods provide highly accurate measures of cell volume and hemoglobin concentration, even from highly nonspherical RBCs^29^, and are even potentially transferrable to a flow cytometry setup^30^, they require expensive instrumentation and highly trained users to operate.

Light scattering is a well-established metrology method that can determine particle size, polydispersity, and refractive index. Thus, with appropriate calibration, it also can measure red cell volume (MCV), distribution width (RDW), and mean cell hemoglobin concentration (MCHC, related to refractive index) of a diluted whole blood sample with no complex flow or sample handling. Previously, our lab developed a mobile phone-based device that used elastic light scattering to accurately measure the shape information of particle suspensions, including blood, with nanometer precision^31^, however in that early study we only focused on extracting the mean size of samples, and studies on blood were limited to a single subject without corresponding clinical measurements. In this report we present two improvements to prior work on anemia diagnosis. First, because there has been only a single study of red cell parameters to diagnose IDA and TT in Chinese children and it did not include information about healthy controls^32^, we collected a historical dataset of 384 children. Using this data we use discriminant function analysis to develop a statistically robust diagnostic index, termed Joint Indicator A, especially designed for use with a Chinese population with performance substantially higher than prior reported indices. This index has an emphasis on those red cell parameters that can be collected by a portable light scattering device. The second improvement is to design a new portable light scattering device that incorporates two wavelengths and an expanded range of collection angles to robustly extract red cell parameters from diluted whole blood samples. We then validate the performance of this instrument against a clinical gold standard, with encouraging initial results.

## Results

### Overview of Historical Data

Table 1 presents the descriptive statistics of the 384 patients analyzed in our retrospective study. P-values were obtained using ANOVA analysis implemented in the statistical software SPSS 13.0. In each case we must perform comparisons between all anemias and healthy patients, as well as comparisons between the two anemia subtypes. To correct for multiple comparisons, testing was implemented in two ways depending on whether the variances in each subgroup were judged by SPSS to be homogenous or inhomogeneous. In the case of homogenous variances, the p-value was computed using the Bonferroni test, while Dunnett’s T3 test was used in the case of unequal variances. Additionally, gender and age were categorical variables, and the statistical significance of their occurrence rates were computed using the Fisher exact test. All tests were performed at an α = 0.05 significance level. There was no statistically significant difference in sex between groups (p > 0.05). There were significant differences in the age and age composition of each group (p < 0.01), reflecting the reality that anemia is most prevalent among infants in China, and decreases with age^33^.

**Table 1:** Baseline Values in HC Group, IDA Group and TT Group (µ ± σ).

Variable	HC	IDA	TT	p
No. of samples	174	164	46	
Age (years)	5.81 ± 3.81	2.09 ± 3.33^a^	4.05 ± 3.58^ab^	<0.0001
Age group (years)				<0.0001
0~0.5 (N,%)	3(1.72)	17(10.36)	10(21.74)	
0.5~2 (N,%)	46(26.44)	119(72.56)	11(23.91)	
2~6 (N,%)	50(28.74)	12(7.32)	13(28.26)	
6~12 (N,%)	68(39.08)	9(5.49)	11(23.91)	
12~17 (N,%)	7(4.02)	7(4.27)	1(2.18)	
Females (N,%)	70 **(**40.22**)**	55**(**33.54**)**	17**(**36.96**)**	
Males (N,%)	104 **(**59.78**)**	109**(**66.46**)**	29**(**63.04**)**	
RBC (10^12^/L)	4.71 ± 0.35	4.48 ± 0.65^a^	5.51 ± 0.57^ab^	<0.0001
HGB (g/L)	130.28 ± 11.06	94.41 ± 16.24^a^	103.80 ± 11.03^ab^	<0.0001
MCV (fl)	83.93 ± 3.71	69.87 ± 9.08^a^	59.13 ± 4.98^ab^	<0.0001
MCH (pg)	27.58 ± 1.20	21.18 ± 3.67^a^	18.89 ± 1.71^ab^	<0.0001
MCHC (g/L)	329.21 ± 6.66	303.81 ± 19.4^a^	319.37 ± 9.27^ab^	<0.0001
RDW (%)	13.42 ± 0.88	17.80 ± 2.91^a^	18.10 ± 2.83^a^	<0.0001

Note: RBC, red blood count; HGB, hemoglobin; MCV, erythrocyte mean corpuscular volume; MCH, mean corpuscular hemoglobin; MCHC, mean corpuscular hemoglobin concentration. Compared with HC group, ^a^ *p* < 0.05; Compared with IDA group, ^b^ *p* < 0.05.

The differences between RBC, HGB, MCV, MCH, MCHC and RDW for anemic patients of any kind versus healthy controls (HC group) were statistically significant. Comparing the IDA and TT groups, the values of RBC, HGB, MCHC in TT group were relatively higher, while the values of MCV and MCH were relatively lower, with the differences being statistically significant (p < 0.05). The results are shown in Table 1. Interestingly, RDW was not significantly different between IDA and TT groups.

### Discriminant function analysis for diagnosis of anemia *via* red cell parameters

**Figure 1:**
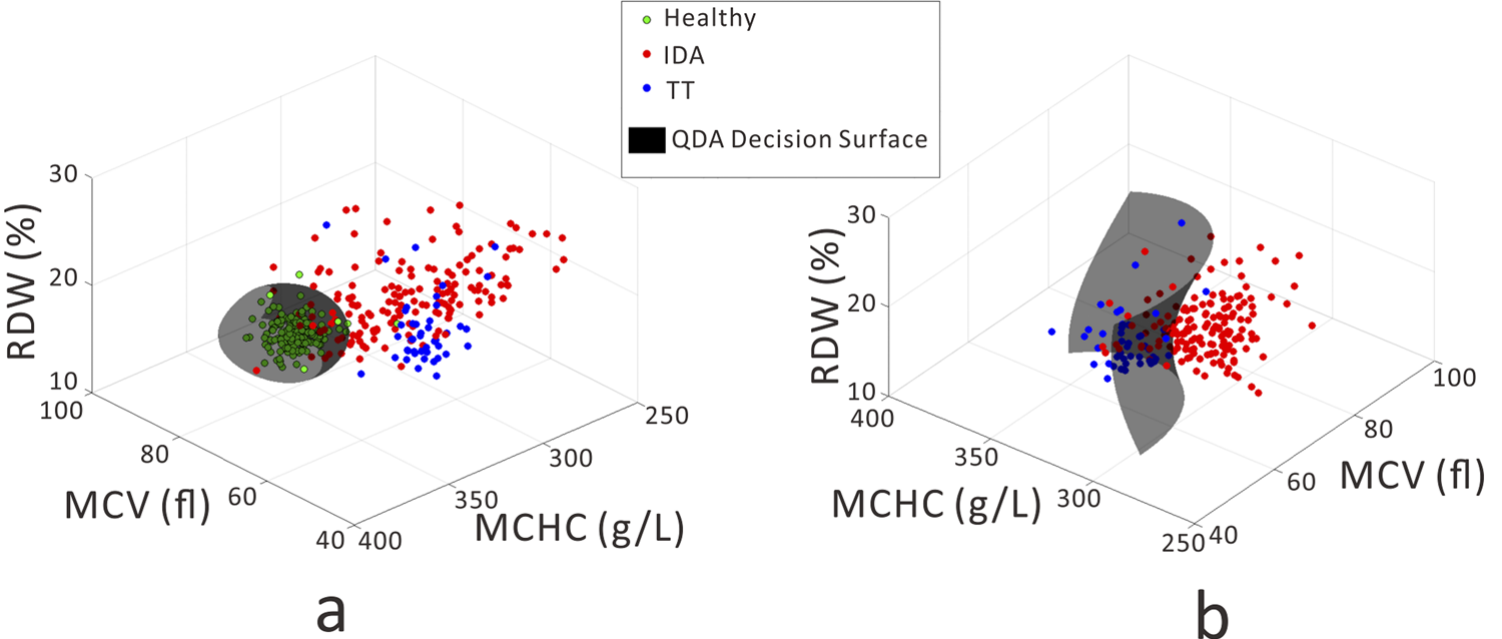
The results of Joint Indicator A based on quadratic discriminant functions. (**a**) Discrimination between healthy and any anemia. (**b**) Discrimination between IDA and TT.

In order to provide statistically robust diagnosis of anemia using red cell parameters, we utilized discriminant function analysis (DFA) to find the best diagnostic index that separates healthy and anemic patients, and then further separates IDA and TT. DFA is an extension of traditional Fisher discriminant analysis for classifiers that are not necessarily linear in shape. In general terms, DFA constructs a decision boundary which provides the best separation between two or more groups, with the shape of the boundary being an Nth order polynomial. In our case, we utilized a quadratic discriminant analysis, which represents a balance between allowing the algorithm to construct a complex decision surface without making that surface so complex as to be susceptible to data overfitting. An advantage of DFA over some other classification algorithms (such as K-nearest neighbors), is that its output is an analytic function that can be easily shared with other users, and whose output can be computed via pen and paper. We also evaluated support vector machines for establishing a diagnostic index, but found that its results were comparable to DFA. Thus, given the simplicity and parameter-free training, we selected DFA as the classification algorithm for this study. Using DFA, we evaluated different combinations of red cell parameters metrics in their ability to separate healthy controls from anemic patients, and to separate IDA and TT. Examples of the quadratic decision surfaces generated by DFA can be seen as the shaded black shapes in Fig. 1. Utilizing one, two, three, four, five, or six red cell parameters, we can compute different discriminant functions and compare their performance. The complete results are available in the supplemental material. In Tables 2 and 3, we summarize the results of the best combination of one, two, three, and six parameters for diagnosing HC vs. anemia of any form, and for diagnosing IDA vs. TT, respectively. Each combination is evaluated through a receiver-operator curve (ROC) on the basis of the area under the curve (AUC), sensitivity, specificity, and Youden’s index^34^ at an optimum cut-off value.

**Table 2:** Results of DFA analysis of different combinations of red cell parameters to separate HC and anemia groups.

	MCH	RDW, MCH	MCH, MCHC, RDW	MCV, MCHC, RDW (JIA)	MCV, MCHC, RDW, MCH, HGB, RBC (JIB)
AUC (%)	96.70	97.13	98.27	97.99	98.01
AUC (95% CI)	(94.95, 98.45)	(95.35, 98.91)	97.14 to 99.4	(96.73, 99.25)	(96.71, 99.31)
cut-off value	0.97	1.32	2.05	1.82	1.38
sensitivity (%)	95.98	95.40	94.25	94.25	94.25
specificity (%)	92.38	95.24	96.67	95.71	95.24
Youden index	0.88	0.91	0.91	0.90	0.89

**Table 3:** Results of DFA analysis of different combinations of red cell parameters to separate IDA and TT.

	MCV	MCV, MCHC	MCV, MCHC, RDW (JIA)	MCV, MCHC, RDW, MCH, HGB, RBC (JIB)
AUC (%)	84.32	94.20	95.32	95.20
AUC (95% CI)	(78.67, 89.97)	(90.8, 97.6)	(92.66, 97.98)	(92.40, 98.00)
cut-off value	−0.19	−0.48	−0.25	−2.26
sensitivity (%)	69.51	90.85	94.00	87.80
specificity (%)	91.30	91.30	90.01	93.48
Youden index	0.61	0.82	0.84	0.81

Our diagnostic procedure first discriminates between HC and anemia of any type. Then the anemic patients are further separated into IDA and TT subgroups. As summarized in Table 2, for discriminating healthy from anemia of any form, the single variable with the largest diagnostic power is MCH (or, similarly, MCHC). Adding additional variables increases the discriminatory power only slightly. For discriminating IDA vs TT, the single variable with the largest discriminatory power is MCV. The best result for discriminating healthy from anemia of any type using three parameters is using MCH, MCHC and RDW. The best result for discriminating IDA and TT using three parameters was obtained with MCV, MCHC, and RDW. We call the discriminant function obtained with these variables “Joint Indicator A,” (JIA). We note that its ability to separate anemic patients from healthy is nearly as high as the optimum MCH/MCHC/RDW combination. Using JIA, we obtained sensitivity and specificity >90% for separating healthy, IDA, and TT subjects. Combining all six possible red cell parameters we obtain another discriminant function that we dub “Joint Indicator B,” (JIB). As is clear from Tables 2 and 3, the performance of JIA is not significantly worse than JIB. This indicates that the three parameters MCV, MCHC, and RDW are an acceptable selection of variables for discriminating IDA and TT from healthy controls, as well as discriminating IDA from TT.

The MCV, MCHC, and RDW values for each patient can be plotted in a three-dimensional space, as shown in Fig. 1. It is clear that in this space, healthy and anemic patients fall into spatially distinct regions. Allowing DFA to generate the best (quadratic) boundary between the two groups produces the decision surface in Fig. 1a. As detailed in the supplemental material, once this decision surface has been computed based on historical training data, new samples can be classified into healthy or anemic based on their location relative to the surface. Figure 1b shows a similar analysis to separate IDA from TT.

### Comparison between JIA and other published anemia diagnostic indices

Currently published anemia diagnostic indices are as follows: (1) red blood cells: RBC^35^; (2) Mentzer index(MI): MCV/RBC^36^; (3) Shine and Lal index(SL): MCV^2^*MCH *0.01^37^; (4) England and Fraser index(EF): MCV-RBC-(5 *Hb)−5.19^38^; (5) Srivastava index(S): MCH/RBC^39^; (6) Green and King index(GK): MCV^2^* RDW/(100 *Hb)^40^; (7) Red blood cell distribution width: RDW^41, 42^; (8) Red blood cell distribution width index(RDWI): MCV*RDW/RBC^43^;(9) Ricera index(R): RDW/RBC^44^; (10) Ehsani index(E): MCV-10*RBC^45^; (11) Sirdah index (Si): MCV-RBC-3*Hb^46^.

**Figure 2:**
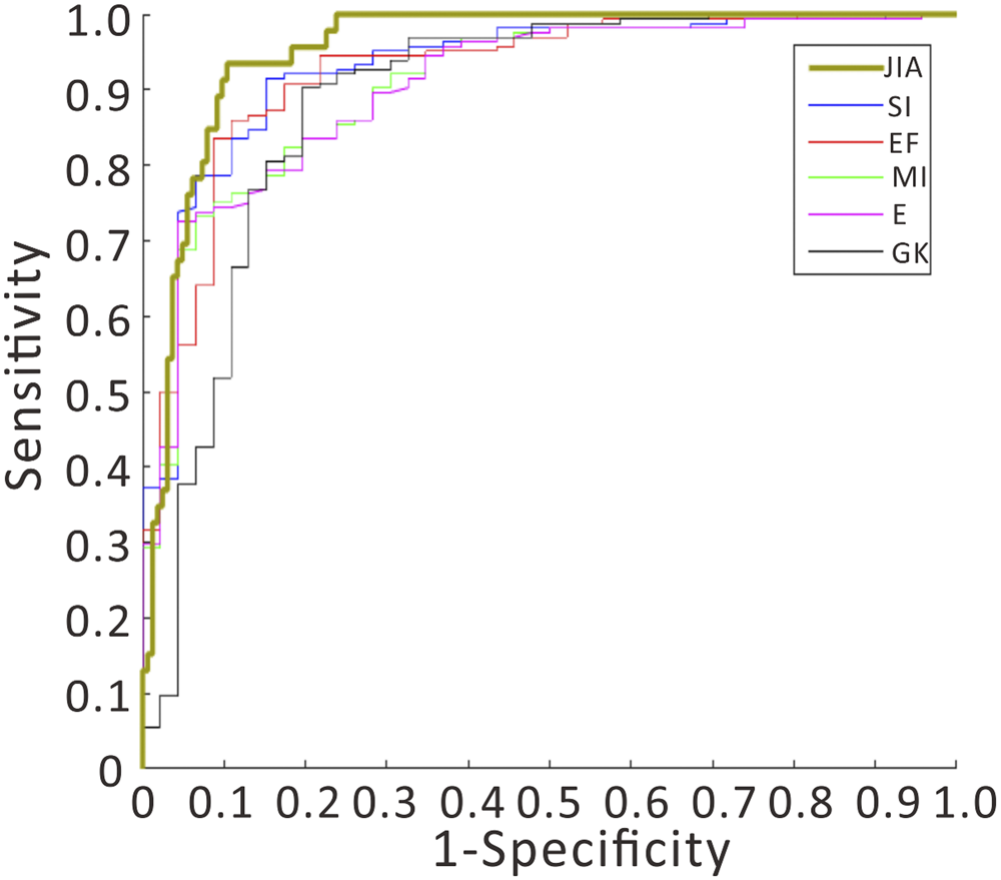
The ROC curves of different diagnostic indices.

Cut-off values for each index are provided in the literature, but clearly differ between different patient populations. To provide the fairest analysis, we calculated new optimum cut-off values for each index, and computed new ROC curves for each index using these optimum cut-off values. The performance comparison between these established indices and JIA is shown in Table 4, with the ROC curves for JIA and the 5 top-performing indices shown in Fig. 2. As is clear from Table 4 and Fig. 2, JIA clearly outperforms previously published metrics, with an AUC of 95.32% and sensitivity and specificity of 94 and 90 percent, respectively. We also note here that our methodology can not only separate IDA and TT, but also separates anemia from healthy controls, which is not straightforward with the other indices.

**Table 4:** The AUC values, cut-off values, sensitivity and specificity of different anemia diagnostic indices.

Index	Published		Our research				
	AUC(%)	Cut-off	AUC(%)	Cut-off	Sens.(%)	Spec.(%)	Youden index
JIA	-----	-----	95.32	−0.25	94.00	90.01	0.84
Si	87.62	27.00	92.90	26.29	91.50	84.80	0.76
EF	88.57	0.00	92.40	3.73	86.00	89.10	0.75
MI	78.57	13.00	90.50	13.21	73.20	93.50	0.67
E	58.10	4.40	90.50	16.45	72.60	95.70	0.68
GK	83.81	72.00	88.60	67.98	90.20	80.40	0.71
RBC	79.52	5.00	88.40	5.18	80.40	83.50	0.63
RDWI	78.10	220.00	87.60	206.80	93.90	73.90	0.67
S	76.19	3.80	83.30	4.10	65.90	91.30	0.57
SL	66.67	904.00	79.40	884.04	61.60	91.30	0.52
R	80.43	3.10	79.00	3.36	79.90	69.60	0.50
RDW	70.90	14.00	51.80	14.85	97.80	15.90	0.13

### Validation of a low-cost, portable diagnostic device based on elastic light scattering

**Figure 3:**
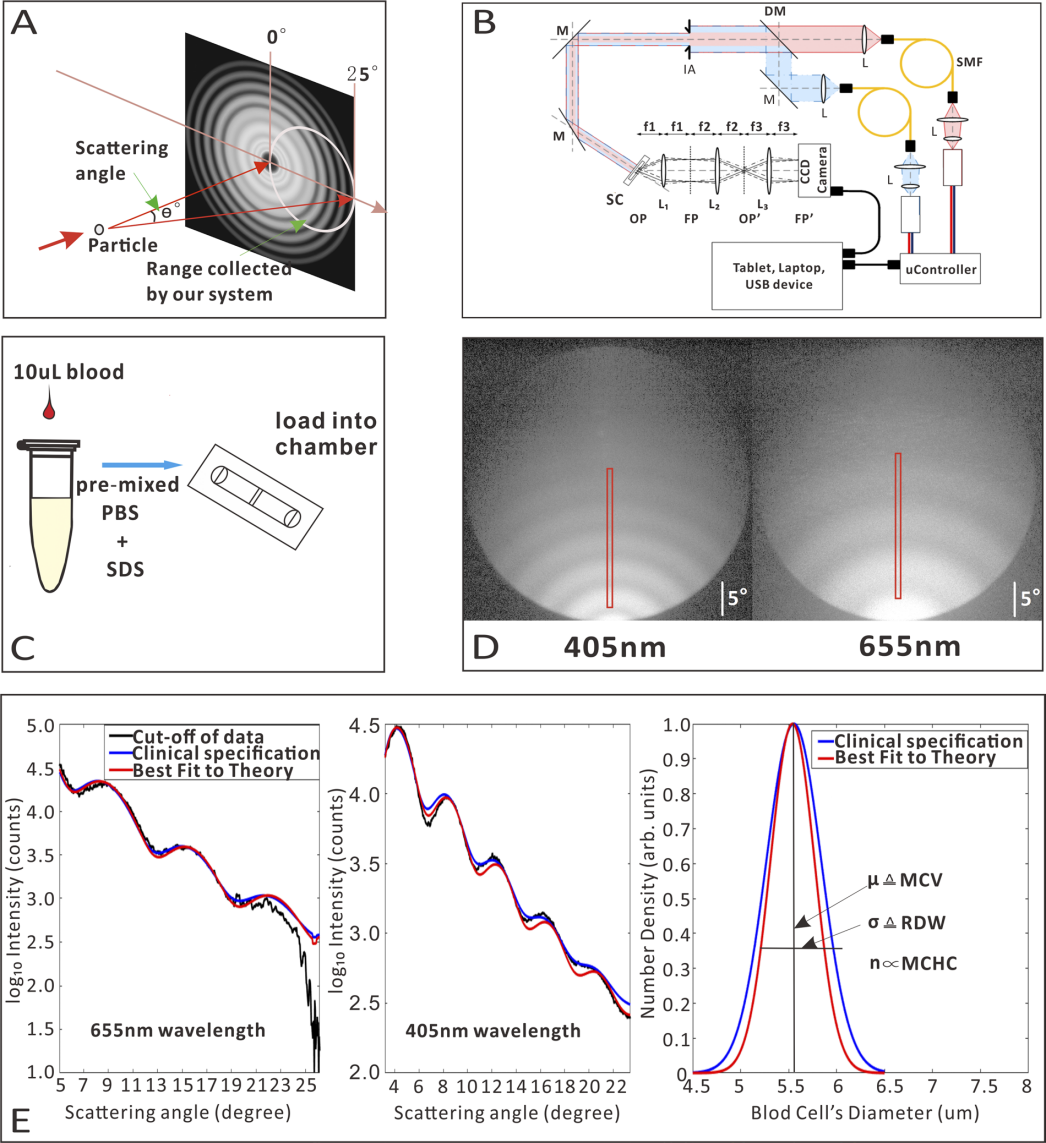
(**A**) Particle scattering model. (**B**) System Schematic. (**C**) Sample preparation. (**D**) Scattering images. (**E**) Data processing. Abbreviations: L = Lenses, SMF = Single mode fiber, M = Mirror, DM = Dichroic Mirror, IA = Iris Aperture, SC = Sample Chamber, OP and OP’ = Object plane and its conjugate, FP and FP’ = Fourier Plane and its conjugate. µ, expected value of the Gaussian distribution; σ, variance of the Gaussian distribution; n, refractive index of the blood.

Based on the results of our historical study, measurement of only the three parameters contained within JIA (MCV, MCHC, and RDW) are needed for a reasonably sensitive and specific diagnosis of anemia. As optical light scattering can provide the mean particle volume, average particle refractive index, and polydispersity of a particle suspension such as blood^47^, we can use such a system to identify MCV, MCHC, and RDW. The basic concept of elastic scattering is shown schematically in Fig. 3A. A single particle is illuminated by a planar wave of monochromatic light, such as from a laser, giving rise to a pattern of scattered light versus different scattering angles. This pattern is highly sensitive to particle shape and size. Several automated analyzers use variations on this basic scattering method to size red cells as they flow past a laser beam one-at-a-time, using the familiar forward and side scattering channels of modern flow cytometers. In our instrument, shown schematically in Fig. 3B, a blood sample is placed in the path of collimated light from red and blue laser diodes (AH-650-1-801 and AH405-201230, respectively, AiXiZ). The laser beams are combined prior to being directed onto the sample by a dichroic mirror (FF458-Di02-25 × 36, Semrock). The blood is held in disposable chambers of 100 micron thickness (C10283, Invitrogen). Scattered light is collected and the pattern of light versus angle is imaged onto a CCD detector (Lu130, Lumenera). The imaging system is constructed such that the CCD is looking not at an image of the red blood cells, but at the Fourier plane of the collection lens, where spatial positions in the Fourier plane correspond to the polar and azimuthal scattering angles in the sample plane. Contrasting with flow-based methods, our instrument records the scattering from >10,000 RBCs in a single shot with no sample flow. Thus, rather than determining size and/or refractive index of a single particle, we determine these parameters for a population of particles simultaneously. For blood measurements, preparation is shown in Fig. 3C. First, blood is collected via a finger stick or venous draw. This blood is then diluted with a mixture of PBS and SDS and then placed in the instrument. Experimental data is collected, representing patterns of scattered intensity versus scattering angle, as shown in Fig. 3D. We note that there is a slightly higher system background in the 655 nm channel (magnified by the log scaling of the image) that we believe is due to stray light from the red laser contributing a small amount of background haze.

A subregion of this image is extracted to form a 1D curve of scattering intensities that can be fit to theory, as shown in Fig. 3E. The details of the experimental fit are provided in the supplemental material, as well as prior reports^31^. The values of the particle size distribution (mean size, distribution width) and refractive index that produce the best agreement between experiment and theory are identical to the blood parameters returned by a standard analyzer. The mean particle size corresponds to the MCV, the distribution width to the RDW, and the average refractive index to the MCHC, as discussed in Friebel and Meinke^48^. The time required for analysis is approximately 4 minutes per sample using MATLAB and a standard laptop, however this is likely to improve in the future, as the code was not written with speed in mind.

**Figure 4:**
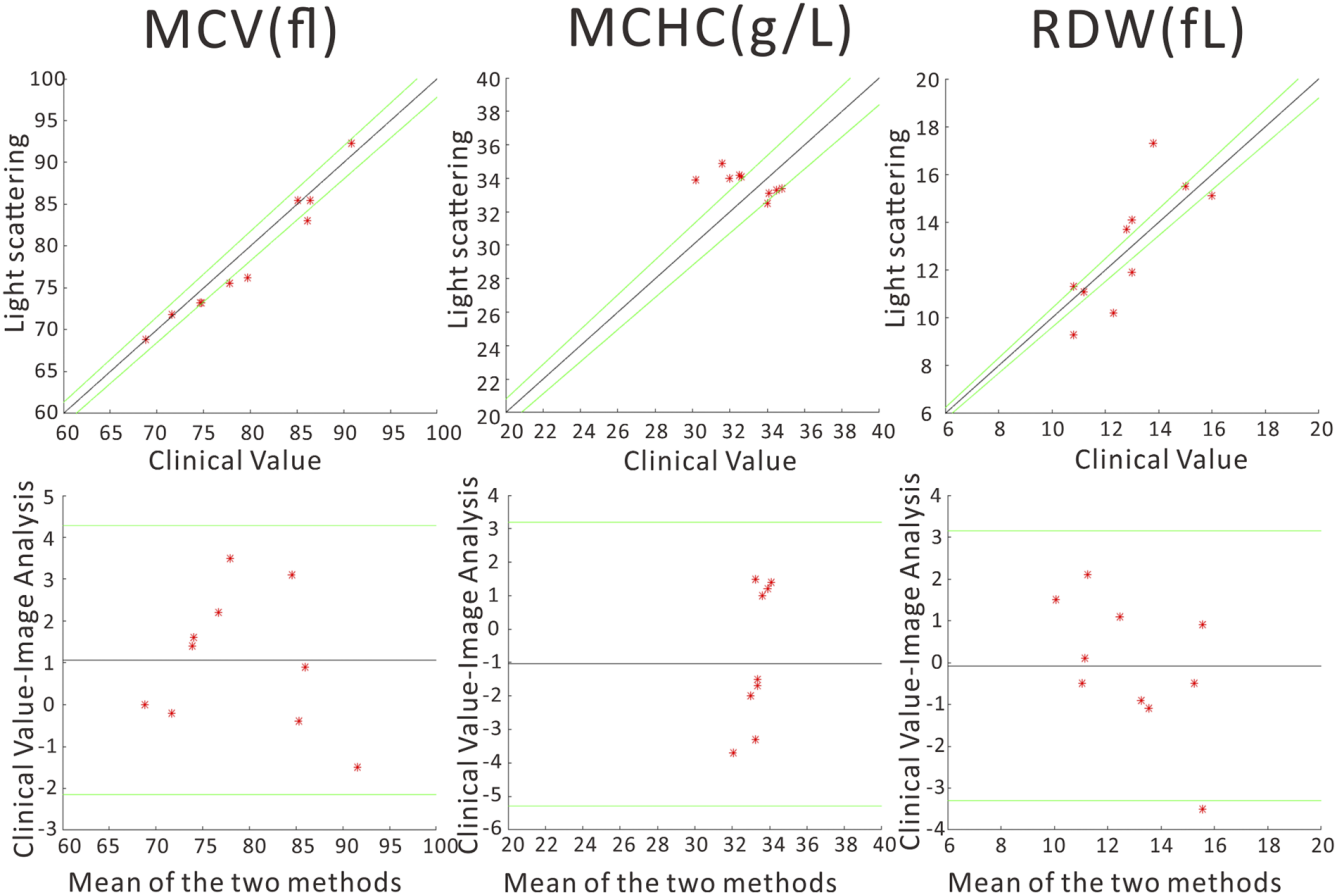
Top row: Correlation between the clinical standard and our instrument. Lines of perfect agreement are shown in black, while the 95% confidence interval of the clinical gold standard are shown in green. Bottom row: Bland-Altman analysis, the black lines depict the bias of our measurements compared to the clinical gold standard, while the green lines are the 95% confidence intervals for the disagreement between the two sets of results.

In order to test the proof of concept of our device, we collected venous blood from 10 discarded blood samples from the University of California Davis Pathology Department’s clinical labs. After measurement of the experimental scattering patterns, the results of the particle size extraction were compared with standard blood cell results obtained from the clinical laboratory’s Beckman-Coulter blood analyzer. The results of this comparison are shown in Fig. 4. The top row shows the correlation between the two devices, while the bottom row presents the results of a Bland-Altmann analysis. The results of our analysis on this small group of blood samples demonstrates reasonable agreement with clinical results, particularly for MCV, where average errors of less than 1 fL are obtained, and overall the error level is similar to that obtained by the clinical gold standard. While there is still good correlation between the clinical value and our device for the other two parameters, MCHC and RDW nevertheless have larger errors that will need to be corrected before full implementation of the method outlined here. These errors we believe are related to a sample-dependent background in our device, whose origin has not been able to be fully determined. While its shape qualitatively matches a possible background caused by platelets (see Supplemental Information), the apparent magnitude of the discrepancy between experiment and theory suggests that other factors may also be contributors, such as stray light, or scattering from other components of blood such as WBCs and plasma protein. Further experiments will be required to fully elucidate this discrepancy. This background impacts the apparent contrast of the Mie fringes, which affects our estimation of MCHC and RDW. A laser with longer wavelength may be used in the future as the scattering from longer wavelengths is less impacted by small scatterers. Introducing a second camera to record backscattering angles may also improve fitting results, as higher angles are more sensitive to subtle changes in cell shape and size^49^. Altering our analysis method may also allow us to estimate and compensate for this background.

## Discussion

In this paper we have presented a retrospective study of red cell parameters in Chinese children suffering from iron deficiency, thalassemia minor, along with healthy controls. Based on this retrospective data we were able to obtain a new diagnostic index using the statistically robust quadratic discriminant analysis. Owing to its strong statistical foundation, our new index has significantly improved performance compared to prior indices. Our planned paradigm for this index is that all patients identified as TT by the low-cost screening method should go to the hospital to get further testing. As these patients will eventually receive the correct diagnosis, even if they were originally healthy or had IDA, the key diagnostic errors are for misdiagnoses outside of this category. Using the sensitivity and specificity of JIA, along with the distribution of the two anemias in Southeast China, the overall proportion of misdiagnosis in a hypothetical general population is very small. Assuming ~20% incidence of IDA and 10% incidence of TT in China^50, 51^, overall 98.5% of population would receive correct diagnosis by our proposed screening method, while minimizing the cost and burden to patient and to the country’s medical infrastructure. Our current study focuses only on anemia diagnosis, without regard to anemia severity. Further study on how RBC parameters vary with anemia severity may help clarify whether our misdiagnoses are due to simple biological variability, or due to variations in anemia severity.

Furthermore, results were optimized by using only three parameters: MCV, MCHC, and RDW. All of these parameters can be obtained from a measurement of elastic light scattering. We then constructed a simple, low-cost light scattering system that does not require tedious multi-step blood preparation or precise sample placement. Therefore, it is appropriate for operation by minimally or untrained users. The proof-of-concept performance of this device was tested on 10 blood samples, with the results showing modest but promising agreement with a clinical gold standard. These preliminary results, along with our high-performing diagnostic index, lay the groundwork for future studies that will demonstrate widespread, cost-effective screening for iron deficiency and Thalassemia minor in rural and low-resource areas in Southeast Asia and other areas where Thalassemia minor is endemic.

## Methods

### Collection of Historical Data and Diagnostic Criteria

In order to generate a statistically robust diagnostic index, historical clinical data was collected from 210 anemic children and 174 healthy children. 164 of the anemic children were diagnosed with IDA and 46 of them were diagnosed with TT. Their blood test reports were all collected by the outpatient department of the Children’s Hospital of Chongqing Medical University from January 2016 to August 2016. Our study was approved by the Ethics Committee of the Children’s Hospital of Chongqing Medical University. WHO diagnostic criteria were used to distinguish different types of anemia^52^. Patients 6 months to 6 years old with hemoglobin less than 110 g/L and those 6 to 14 years old with hemoglobin less than 120 g/L were considered anemic. For diagnosis of IDA, the serum iron of patients must be less than 11 umol/L. For deletion α-thalassemia patients, PCR-RDB technology was used to detect the −α3.7, −α4.2 and –SEA α-thalassemia deletion genes. For mutation α-thalassemia patients, PCR-RDB technology was used to detect the QS, CS and WS common mutation sites. For β-thalassemia patients, PCR-RDB technology was used to detect the Beta-globin genes as following common mutation sites and start codons: CD41-42(-TCTT), IVS-2-654 C → T, CD17 A → T, −28 A → G, CD26 G → A, CD71-72(+A), CD43 G → T, −29 A → G. PRC-RDB was also used to identify the following nine rare mutation sites: ATG → AGG, CD14-15(+G), CD27-28(+C), −32 C → A, −30 T → C, IVS-1-1 G → T, IVS-1-5 G → C, CD31(-C), CAP + 40- + 43 (-AAAC).

Fasting venous blood samples were obtained and stored in a Vacutainer ethylene diamine tetra-acetic acid (EDTA) anticoagulation tube. Hematological analysis was done by a Sysmex XE-2100 hematology analyzer with its supporting reagents. The serum iron was measured by the fully automatic biochemical analyzer (VITROS 5.1 FS, Johnson & Johnson Corporation, USA) with its supporting reagents. Thalassemia genetic testing utilized a PCR amplifier (Verity, ABI Corporation, USA), HB-1000 Hybridizer (UVP Corporation, USA), electrophoresis and gel imaging systems (Bio-Rad Corporation, USA), α-thalassemia point mutation gene detection kit (Yaneng BIO Corporation, China) and β-thalassemia point mutation gene detection kit (Yaneng BIO Corporation, China). The procedure was strictly operated in accordance with the instrument and kit instructions by staff in the Children’s Hospital of Chongqing Medical University.

### Data Analysis

All data analysis was performed using MATLAB 2015b (The MathWords, Natick, MA). Discriminant function analysis was implemented *via* MATLAB’s canned “classify” function. All other analysis, including Mie theory analysis and data fitting (described in complete detail in the Supplemental Information) was performed using MATLAB scripts developed in-house.

### Validation of Portable Red Cell Analyzer

For testing of our portable red cell analyzer, we measured ten adult subjects’ blood on our device along with paired measurements on a Beckman Coulter clinical analyzer at the University of California, Davis (UC Davis) Medical Center. All samples in the experimental study were anonymized, discarded blood samples that had been stripped of all identifying information, and thus were waived from requiring IRB approval. Each sample was stored in EDTA coated tubes which prevent coagulation of the blood. Sample preparation steps for measurement in on our device (described in detail in Section 3.4) are as follows: To provide sphering and dilution, 10 µL of whole blood is diluted 300 times in phosphate buffered saline (PBS) containing 10 µL sodium dodecyl sulfate (SDS). SDS is an anionic surfactant which will work on the surface tension of blood cells and force them into a uniform spherical shape. A similar reaction occurs within the measurement path of commercial automated analyzers^31^. 10 µL of this sample is then placed within a commercially available disposable sample chamber (Life Technologies, C10228) and measured and analyzed as described in Section 3.4 and the Supplemental Material.

### Statement on Human Subjects Testing

As described above, for our retrospective data collection conducted at the Children’s Hospital of Chongqing Medical University, our study was approved by the Ethics Committee of the Children’s Hospital of Chongqing Medical University. Because all reports were anonymized, informed consent was not required. For experimental data collection conducted at the University of California, Davis Medical Center, all samples were anonymized, discarded blood samples that had been stripped of all identifying information, and thus were waived from requiring informed consent or IRB approval. Methods were carried out in accordance with relevant guidelines and regulations.

### Data Availability

The datasets generated during and/or analyzed during the current study are available from the corresponding author on reasonable request.

## Electronic supplementary material

Supplemental Information
