# Supplementary material for: A new red cell index and portable RBC analyzer for screening of iron deficiency and Thalassemia minor in a Chinese population
Source: Sci Rep. 2017 Sep 5;7:10510. doi: 10.1038/s41598-017-11144-w (PMC5585383; doi:10.1038/s41598-017-11144-w)
Supplement: Supplementary file 1 — Supplemental Information [file 41598_2017_11144_MOESM1_ESM.pdf]

Supplemental Material for "A new red cell index and portable RBC analyzer for screening of iron deficiency and Thalassemia minor in a Chinese population"

Lieshu Tong[1], Josef Kauer[2,3], Sebastian Wachsmann-Hogiu[3,4,5], Kaiqin Chu[1], Hu Dou[6,*], Zachary J. Smith[1,*]

[1]University of Science and Technology of China, Department of Precision Machinery and Precision Instrumentation, Hefei, Anhui, China

[2]Beuth Hochschule für Technik Berlin, Berlin, Germany

[3]Center for Biophotonics Science and Technology, University of California, Davis, Sacramento, CA, USA

[4]University of California, Davis, Department of Pathology & Laboratory Medicine, Sacramento, CA, USA

[5]McGill University, Department of Bioengineering, Montreal, Quebec, Canada

[6]Department of Clinical laboratory, Ministry of Education Key Laboratory of Child Development and Disorders; Key Laboratory of Pediatrics in Chongqing; Chongqing International Science and Technology Cooperation Center for Child Development and Disorders; Children's Hospital of Chongqing Medical University, Chongqing, China

*Email: 375009808@qq.com (HD), zsmith@ustc.edu.cn (ZJS)

Contents

1. Results of QDA analysis of different combinations of RBC parameters 2

2. Discriminant function for QDA based on JIA 9

3. Physical instrument, and description of the scattering measurement processing flow. 10

1. Results of QDA analysis of different combinations of RBC parameters

All possible combinations of RBC, HGB, MCV, MCH, MCHC and RDW analyzed via QDA for separating healthy and anemia and separating IDA and TT are as follows:

Table 1. Results of ROC curve analysis of different combinations of MCV, MCHC, RDW and MCH in HC and anemia groups

	RBC	HGB	MCV	MCH	MCHC	RDW	RBC,HGB	RBC,MCV
AUC（%）	78.09	95.48	94.30	96.70	88.15	93.90	97.13	96.40
AUC (95% CI)	73.51 to 82.66	93.34 to 97.62	91.79 to 96.8	94.95 to 98.45	84.66 to 91.64	91.3 to 96.4	95.48 to 98.79	94.5 to 98.4
cut-off value	0.13	0.39	0.79	0.97	0.40	0.62	0.95	0.78
sensitivity（%）	86.78	93.68	95.40	95.98	91.95	95.40	94.25	94.83
specificity（%）	59.05	91.90	89.52	92.38	75.71	88.10	94.29	94.29
Youden index	0.46	0.86	0.85	0.88	0.68	0.83	0.89	0.89

	RBC,MCH	RBC,MCHC	RBC,RDW	HGB,MCV	HGB,MCH	HGB,MCHC	HGB,RDW	MCV,MCH
AUC（%）	97.59	93.96	96.20	97.26	97.56	96.21	97.41	97.40
AUC (95% CI)	96.16 to 99.03	91.67 to 96.24	94.4 to 98	95.62 to 98.91	96.06 to 99.06	94.29 to 98.13	95.91 to 98.91	96 to 98.8
cut-off value	0.82	0.57	1.09	0.91	1.30	1.06	1.12	1.59
sensitivity（%）	94.25	90.80	92.53	94.83	94.25	91.38	93.68	93.68
specificity（%）	94.76	85.71	91.90	94.76	95.24	95.71	95.24	94.29
Youden index	0.89	0.77	0.84	0.90	0.89	0.87	0.89	0.88

	MCV,MCHC	MCV,RDW	MCH,MCHC	MCH,RDW	MCHC,RDW	RBC,HGB,MCV	RBC,HGB,MCH	RBC,HGB,MCHC
AUC（%）	97.70	96.90	97.98	97.13	95.92	97.41	97.35	97.30
AUC (95% CI)	96.4 to 99	95.1 to 98.8	96.8 to 99.16	95.35 to 98.91	93.9 to 97.93	95.88 to 98.94	95.78 to 98.92	95.7 to 98.9
cut-off value	1.62	1.20	1.71	1.32	0.43	0.64	1.95	2.10
sensitivity（%）	94.25	95.40	94.25	95.40	95.40	95.40	93.10	93.68
specificity（%）	94.76	94.29	95.24	95.24	87.62	93.81	95.24	96.19
Youden index	0.89	0.90	0.89	0.91	0.83	0.89	0.88	0.90

	RBC,HGB,RDW	RBC,MCV,MCH	RBC,MCV,MCHC	RBC,MCV,RDW	RBC,MCH,MCHC	RBC,MCH,RDW	RBC,MCH,RDW	HGB,MCV,MCH
AUC（%）	97.73	97.24	97.44	97.69	97.77	98.04	96.64	97.40
AUC (95% CI)	96.26 to 99.2	95.57 to 98.91	95.84 to 99.04	96.14 to 99.25	96.35 to 99.18	96.71 to 99.37	94.92 to 98.36	95.9 to 98.9
cut-off value	-0.01	0.96	1.19	1.57	1.21	-0.18	0.73	0.76
sensitivity（%）	95.98	93.10	93.68	94.83	93.68	95.40	92.53	92.53
specificity（%）	94.29	95.71	95.71	96.67	95.71	94.76	91.90	95.24
Youden index	0.90	0.89	0.89	0.91	0.89	0.90	0.84	0.88

	HGB,MCV,MCHC	HGB,MCV,RDW	HGB,MCH,MCHC	HGB,MCH,RDW	HGB,MCHC,RDW	MCV,MCH,MCHC	MCV,MCH,RDW	MCV,MCHC,RDW
AUC（%）	97.49	97.98	97.65	98.04	97.60	97.24	97.74	97.99
AUC (95% CI)	95.91 to 99.06	96.6 to 99.37	96.13 to 99.17	96.71 to 99.38	96.1 to 99	95.77 to 98.7	96.41 to 99.07	96.73 to 99.25
cut-off value	1.28	1.72	2.01	2.17	1.51	2.49	1.65	1.82
sensitivity（%）	93.68	94.25	93.68	93.68	93.68	92.53	93.10	94.25
specificity（%）	95.71	97.14	96.19	96.67	95.24	95.24	95.71	95.71
Youden index	0.89	0.91	0.90	0.90	0.89	0.88	0.89	0.90

	MCH,MCHC,RDW	RBC,HGB, MCV,MCH	RBC,HGB, MCV,MCHC	RBC,HGB, MCV,RDW	RBC,HGB, MCH,MCHC	RBC,HGB, MCH,RDW	RBC,HGB, MCHC,RDW	RBC,MCV, MCH,MCHC
AUC（%）	98.27	97.56	97.90	97.90	97.52	97.90	97.73	96.99
AUC (95% CI)	97.14 to 99.4	96.07 to 99.05	96.51 to 99.29	96.54 to 99.25	95.98 to 99.06	96.51 to 99.28	96.21 to 99.26	95.31 to 98.67
cut-off value	2.05	1.42	1.34	1.71	2.63	-0.01	2.95	2.03
sensitivity（%）	94.25	93.10	94.25	94.83	92.53	95.40	93.10	91.95
specificity（%）	96.67	94.76	95.24	96.19	96.19	94.76	97.14	95.71
Youden index	0.91	0.88	0.89	0.91	0.89	0.90	0.90	0.88

	RBC,MCV MCH,RDW	RBC,MCV, MCHC,RDW	RBC,MCH, MCHC,RDW	HGB,MCV, MCH,MCHC	HGB,MCV, MCH,RDW	HGB,MCV, MCHC,RDW	HGB,MCH, MCHC,RDW	MCV,MCH, MCHC,RDW
AUC（%）	97.75	97.74	97.97	96.94	97.90	97.88	97.88	97.74
AUC (95% CI)	96.21 to 99.29	96.18 to 99.3	96.58 to 99.36	95.21 to 98.66	96.5 to 99.3	96.41 to 99.35	96.41 to 99.35	96.43 to 99.04
cut-off value	-1.77	2.35	0.93	2.91	-0.47	1.28	1.28	2.91
sensitivity（%）	98.28	93.10	94.83	91.95	95.98	93.68	93.68	92.53
specificity（%）	91.43	97.14	96.19	96.19	93.33	96.67	96.19	96.67
Youden index	0.90	0.90	0.91	0.88	0.89	0.90	0.90	0.89

	RBC,HGB,MCV,MCH,MCHC	RBC,HGB,MCV,MCH,MCHC	RBC,HGB,MCV,MCHC,RDW	RBC,HGB,MCH,MCHC,RDW	RBC,MCV,MCH,MCHC,RDW	RBC,MCV,MCH,MCHC,RDW	RBC,HGB,MCV,MCH,MCHC,RDW
AUC（%）	97.70	97.94	98.19	97.90	97.40	97.43	98.01
AUC (95% CI)	96.2 to 99.1	96.56 to 99.31	96.92 to 99.46	96.47 to 99.33	95.82 to 98.99	95.88 to 98.99	96.71 to 99.31
cut-off value	1.65	0.41	0.35	0.90	0.88	1.46	1.38
sensitivity（%）	93.68	95.98	95.98	94.83	93.68	93.10	94.25
specificity（%）	94.29	95.24	94.76	95.71	95.24	96.19	95.24
Youden index	0.88	0.91	0.91	0.91	0.89	0.89	0.89

Table 2. Results of ROC curve analysis of different combinations of MCV, MCHC, RDW and MCH in IDA group and TT group

	RBC	HGB	MCV	MCH	MCHC	RDW	RBC,HGB	RBC,MCV
AUC（%）	88.42	73.75	84.32	78.12	76.77	51.50	89.80	90.46
AUC (95% CI)	82.74 to 94.1	66.18 to 81.31	78.67 to 89.97	71.43 to 84.8	70.08 to 83.46	42.6 to 60.5	84.6 to 95.1	85.53 to 95.39
cut-off value	-0.53	-0.43	-0.19	-0.11	0.30	0.12	-0.34	-0.21
sensitivity（%）	83.54	70.73	69.51	56.10	51.83	15.85	82.32	80.49
specificity（%）	80.43	69.57	91.30	91.30	93.48	97.83	86.96	91.30
Youden index	0.64	0.40	0.61	0.47	0.45	0.14	0.69	0.72

	RBC,MCH	RBC,MCHC	RBC,RDW	HGB,MCV	HGB,MCH	HGB,MCHC	HGB,RDW	MCV,MCH
AUC（%）	90.02	91.85	90.11	92.80	89.48	80.77	75.62	94.45
AUC (95% CI)	84.8 to 95.24	87.26 to 96.45	85.63 to 94.59	88.46 to 97.15	84.26 to 94.69	74.55 to 86.99	68.4 to 82.83	91.19 to 97.7
cut-off value	-0.54	-0.59	-0.52	-0.71	-0.65	0.12	-0.19	-0.60
sensitivity（%）	84.15	88.41	84.76	89.63	83.54	61.59	67.68	90.85
specificity（%）	86.96	89.13	80.43	89.13	84.78	91.30	73.91	89.13
Youden index	0.71	0.78	0.65	0.79	0.68	0.53	0.42	0.80

	MCV,MCHC	MCV,RDW	MCH,MCHC	MCH,RDW	MCHC,RDW	RBC,HGB,MCV	RBC,HGB,MCH	RBC,HGB,MCHC
AUC（%）	94.20	89.30	93.13	81.06	81.13	94.96	89.22	94.40
AUC (95% CI)	90.8 to 97.6	84.6 to 94	89.32 to 96.95	74.78 to 87.34	74.8 to 87.46	91.84 to 98.09	83.96 to 94.49	91.1 to 97.7
cut-off value	-0.48	-0.59	-0.57	-0.23	-0.50	-0.52	-1.76	-0.64
sensitivity（%）	90.85	80.49	90.24	62.80	78.66	90.24	82.32	91.46
specificity（%）	91.30	84.78	91.30	89.13	71.74	91.30	84.78	89.13
Youden index	0.82	0.65	0.82	0.52	0.50	0.82	0.67	0.81

	RBC,HGB,RDW	RBC,MCV,MCH	RBC,MCV,MCHC	RVC,MCV,RDW	RBC,MCH,MCHC	RBC,MCH,RDW	RBC,MCH,RDW	HGB,MCV,MCH
AUC（%）	92.00	95.00	94.47	91.70	94.37	91.44	94.20	94.80
AUC (95% CI)	88.2 to 95.9	91.9 to 98.1	91.13 to 97.82	87.4 to 95.9	90.9 to 97.84	87.28 to 95.59	91 to 97.4	91.6 to 98
cut-off value	0.32	-0.22	-0.44	-0.14	-0.84	0.14	-0.21	-1.24
sensitivity（%）	75.61	87.20	90.24	80.49	91.46	76.83	89.02	91.46
specificity（%）	93.48	93.48	91.30	91.30	89.13	91.30	86.96	89.13
Youden index	0.69	0.81	0.82	0.72	0.81	0.68	0.76	0.81

	HGB,MCV,MCHC	HGB,MCV,RDW	HGB,MCH,MCHC	HGB,MCH,RDW	HGB,MCHC,RDW	MCV,MCH,MCHC	MCV,MCH,RDW	MCV,MCHC,RDW
AUC（%）	94.50	93.50	93.39	89.20	84.55	93.92	95.24	95.32
AUC (95% CI)	91.2 to 97.8	90 to 97	89.69 to 97.08	84.7 to 93.8	79.03 to 90.07	90.37 to 97.46	92.55 to 97.93	92.66 to 97.98
cut-off value	-0.53	-0.59	-0.53	0.30	-0.05	-2.05	-0.32	-0.25
sensitivity（%）	90.24	88.41	89.63	72.56	68.29	85.98	89.63	94.00
specificity（%）	91.30	89.13	91.30	91.30	89.13	91.30	91.30	90.01
Youden index	0.82	0.78	0.81	0.64	0.57	0.77	0.81	0.84

	MCH,MCHC,RDW	RBC,HGB, MCV,MCH	RBC,HGB, MCV,MCHC	RBC,HGB, MCV,RDW	RBC,HGB, MCH,MCHC	RBC,HGB, MCH,RDW	RBC,HGB, MCHC,RDW	RBC,MCV, MCH,MCHC
AUC（%）	94.05	94.20	94.40	95.70	93.90	90.32	95.67	94.47
AUC (95% CI)	90.97 to 97.12	90.5 to 97.9	90.8 to 98	93.1 to 98.2	89.9 to 97.9	85.79 to 94.86	93.1 to 98.23	91.13 to 97.82
cut-off value	0.03	-1.67	-0.84	0.11	-1.07	-0.89	-0.06	-2.38
sensitivity（%）	86.59	90.24	90.24	87.80	87.20	75.61	89.63	87.20
specificity（%）	93.48	91.30	93.48	95.65	93.48	91.30	93.48	91.30
Youden index	0.80	0.82	0.84	0.83	0.81	0.67	0.83	0.78

	RBC,MCV MCH,RDW	RBC,MCV, MCHC,RDW	RBC,MCH, MCHC,RDW	HGB,MCV, MCH,MCHC	HGB,MCV, MCH,RDW	HGB,MCV, MCHC,RDW	HGB,MCH, MCHC,RDW	MCV,MCH, MCHC,RDW
AUC（%）	95.72	95.85	95.74	94.40	95.70	95.73	94.20	94.70
AUC (95% CI)	93.18 to 98.26	93.39 to 98.31	93.24 to 98.25	91 to 97.7	93.2 to 98.2	93.23 to 98.23	91.2 to 97.3	91.6 to 97.8
cut-off value	-0.44	-0.47	-0.29	-2.36	-0.63	-0.57	-0.40	-0.79
sensitivity（%）	89.63	91.46	90.24	87.80	89.02	91.46	89.63	81.71
specificity（%）	91.30	93.48	93.48	91.30	91.30	93.48	93.48	95.65
Youden index	0.81	0.85	0.84	0.79	0.80	0.85	0.83	0.77

	RBC,HGB,MCV, MCH,MCHC	RBC,HGB,MCV, MCH,MCHC	RBC,HGB,MCV, MCHC,RDW	RBC,HGB,MCH, MCHC,RDW	RBC,MCV,MCH, MCHC,RDW	RBC,MCV,MCH, MCHC,RDW	RBC,HGB,MCV, MCH,MCHC,RDW
AUC（%）	94.33	95.35	95.40	95.10	95.20	95.25	95.20
AUC (95% CI)	90.67 to 97.98	92.71 to 97.98	92.80 to 98.10	92.3 to 97.9	92.30 to 98.10	92.47 to 98.04	92.40 to 98.00
cut-off value	-2.54	-1.40	0.23	-1.34	-1.40	-1.57	-2.26
sensitivity（%）	85.98	90.24	87.80	89.02	84.76	84.76	87.80
specificity（%）	93.48	91.30	95.65	91.30	93.48	93.48	93.48
Youden index	0.79	0.82	0.83	0.80	0.78	0.78	0.81

2. Discriminant function for QDA based on JIA

QDA functions similarly to other red cell indices, in that the parameters MCV, MCHC, and RDW are entered into an equation and a number is computed. In our case, as discussed in the text, the cutoff value for our function to discriminate between healthy and disease is 1.82 and to discriminate between IDA and TT the discriminant function cutoff is -0.25. Using Joint Indicator A (MCV, RDW, and MCHC), discrimination function between healthy and any anemia utilizes the following function

$$JIA_1 = K_1 + [\text{MCV MCHC RDW}]*L_1 + [\text{MCV MCHC RDW}]*Q_1*\begin{bmatrix}\text{MCV}\\ \text{MCHC}\\ \text{RDW}\end{bmatrix}, \quad \textbf{(Equation S1)}$$

where:

K_1= -2363.29396035386

$$L_1=\begin{bmatrix}10.7140187338230\\ 9.61722890266897\\ 48.7869117745025\end{bmatrix}$$

$$Q_1=\begin{bmatrix}-0.0337311136483862 & -0.00570686254641639 & -0.0431072257922396\\ -0.00570686254641639 & -0.0117007351339145 & -0.0346101909998732\\ -0.0431072257922396 & -0.0346101909998732 & -0.708929001307668\end{bmatrix}.$$

Note that MVC is measured in fL, MCHC is measured in g/L, and RDW is measured in %.

Similarly, the discrimination function between IDA and TT is calculated using Equation S2, identical in form to Eq. S1,

$$JIA_2 = K_2 + [\text{MCV MCHC RDW}]*L_2 + [\text{MCV MCHC RDW}]*Q_2*\begin{bmatrix}\text{MCV}\\ \text{MCHC}\\ \text{RDW}\end{bmatrix}, \quad \textbf{(Equation S2)}$$

except that the values of K_2, L_2, and Q_2 are:

K_2=399.5625

$$L_2 = \begin{bmatrix}-1.32877370057470\\ -2.48262241968588\\ 4.74591216693049\end{bmatrix}$$

$$Q_2 = \begin{bmatrix}0.0110027633046597 & 0.000957005548149722 & -0.00974784016388019\\ 0.000957005548149722 & 0.00375307542895284 & -0.00355479566769303\\ -0.00974784016388019 & -0.00355479566769303 & -0.0380428793187491\end{bmatrix}.$$

To illustrate the method of utilization of our discriminant, we consider three cases chosen from our retrospective dataset. Patient A has an MCV of 91, MCHC of 323 and RDW of 12.6. Patient B has an MCV of 73, MCHC of 300 and RDW of 19.3. Patient C has an MCV of 56.7, MCHC of 319 and RDW of 17.5.

Using Equation S1 above, we can calculate that for determination of Healthy vs. any anemia, Patient A's value is JIA_1 = 4.10, while Patient B and C are -23.51 and -28.78, respectively. Thus, Patient A is classified as healthy, while patients B and C continue on to JIA_2 to determine IDA vs. TT. Using Equation S2, their values of JIA_2 are 4.90 and -3.46. Thus, Patient B is classified as IDA, while patients C is classified as TT.

3. Physical instrument, and description of the scattering measurement processing flow.

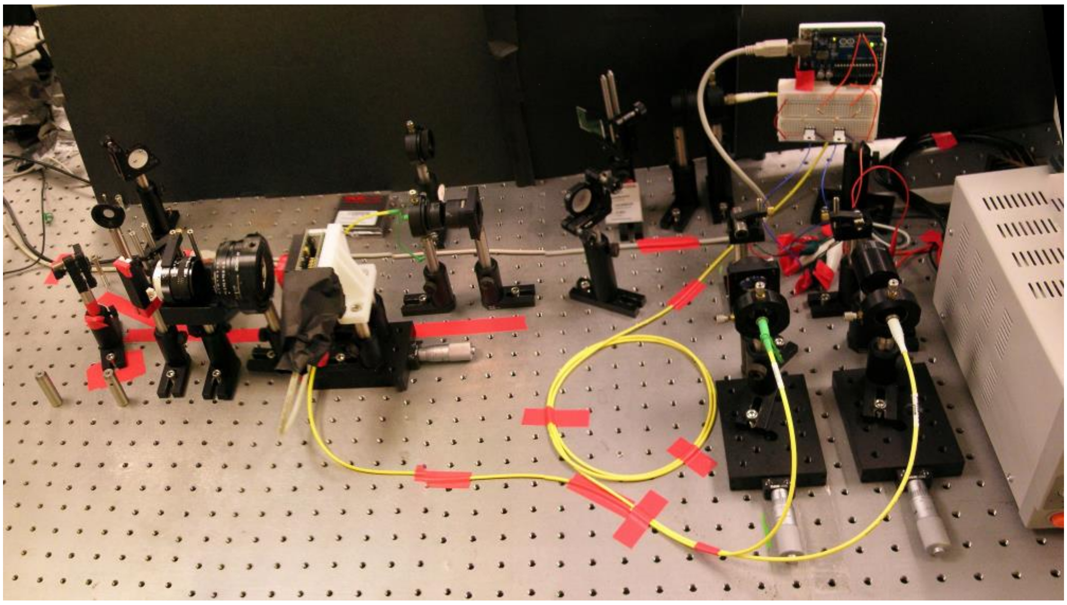

Figure S1 – Actual system.

Our as-built prototype is shown in Figure S1. Laser sources (image right) are coupled into single mode fibers, and then combined and directed by a series of mirrors onto the sample (image left), then imaged onto a board-level CCD camera. We note that the instrument as currently built is composed primarily of empty space, and thus future iterations are expected to be substantially smaller, enabling portable testing.

As described in the main text, this system acquires images of the scattered intensity versus angle. These are then analyzed via a custom analysis routine to extract the RBC parameters through

comparison with Mie theory. Here we describe the detailed data processing required to extract these parameters.

The purpose of our analysis process is to find the best fit between the theoretical scattering patterns and experimental data. In order to achieve this goal, the first important thing is to setup a database of theoretical Mie scattering patterns for a certain range of sizes for both laser wavelengths. This database provides the scattering from red blood cells over a wide size range and refractive index range, across two wavelengths. We used in-house MATLAB scripts to generate the theoretical scattering curves based on established Mie theory. In order to make our calculation process more efficient, we need to set an optimal search range and interval for the size. Normal red blood cells have about 5.5 μm as a spherical diameter, so we choose a size range from 3.5μm to 6.5 μm with 1 nm step resolution. As shown in Figure 3E in the main text, each pixel in the recorded image has an associated angle value. The angle range for the theoretical calculations is exactly the angles measured by our experimental system. The refractive index of the spheres is another important parameter for the calculation. As discussed by Friebel and Meinke in 2006 (ref 45 in the main text), the refractive index of blood is linearly related to the MCHC through the following equation:

$$n_{Hb}(\lambda, c_{Hb}) = n(\lambda)[\beta(\lambda)c_{Hb} + 1] \qquad \textbf{(Equation S3)}$$

where n_{Hb} is the wavelength and concentration dependent refractive index for hemoglobin for the given wavelength λ, and c_{Hb} represents the MCHC. The function n(λ) is the refractive index of water at a given wavelength, and β(λ) is a wavelength-dependent refractive increment tabulated by Friebel and Meinke. For our theoretical database, we calculated theoretical scattering from blood cells with MCHC values in the range from 200 g/L to 400 g/L with 1 g/L steps. Then, we can calculate a 3D matrix $T_{\lambda}(\mathrm{r},\ c_{Hb}(\lambda),\ \theta)$ of theoretical Mie scattering patterns for the two wavelengths, resulting in a 3001 ×201 ×570 matrix theoretical Mie scattering data, where r is the sphere radius. The processing flowchart for comparison between experiment and theory is laid out in Figure S2. The theoretical data contained 3001×201 Mie scattering curves based on 3001×201 different size and refractive index pairs which the particle size corresponds to the MCV, and the refractive index to the MCHC, as discussed in Friebel and Mieinke. For each experimental measurement, a subregion of the raw images (see Fig. 3D) is extracted and averaged to form a 1D curve of scattering intensities versus scattering angle that can be fit to theoretical database. By varying the size value and refractive index value, we want to find the best particle size distribution

whose theoretical scattering most closely matches the experimental scattering pattern.

To start the fitting process, we first determine the mean cell volume and MCHC by comparing the height and position of the first two peaks of the experimental data. Step 1 is to find the exact

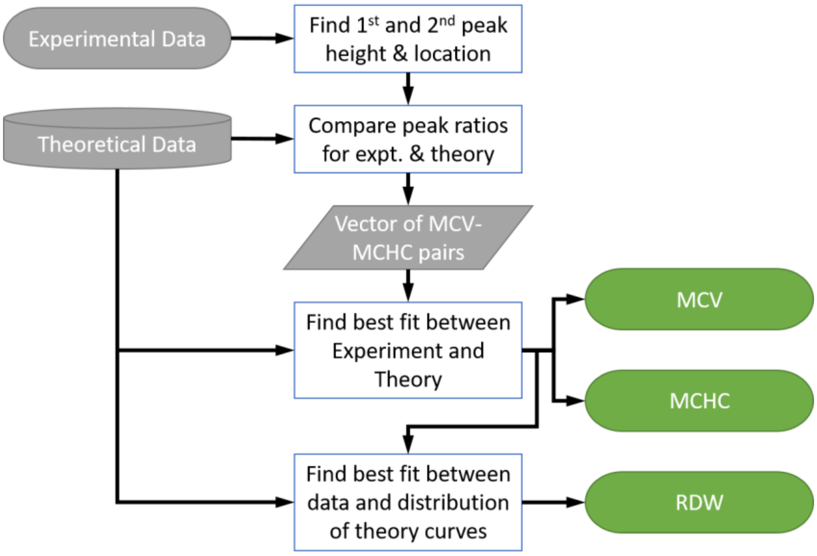

Figure S2 – Fitting process between experiment and theory. Gray elements are input data, blue boxes are analytical operations, and green elements are output red cell parameters.

angular position of the first two maxima of the scattering curve. Because of noise and pixilation of our detector, we fit a small region around each maximum to a 5th order polynomial. The location and maximum intensity of each maximum is then determined for both the experimental data and theoretical data, as shown in Figure S3A. We can compute the ratio of these intensities for the experimental data and for the theoretical database. However, due to experimental discrepancies this method works best with the 405 nm illumination wavelength, and has larger errors with the 655nm wavelength. The reason for this additional error in the 655nm wavelength is simply due to the fact

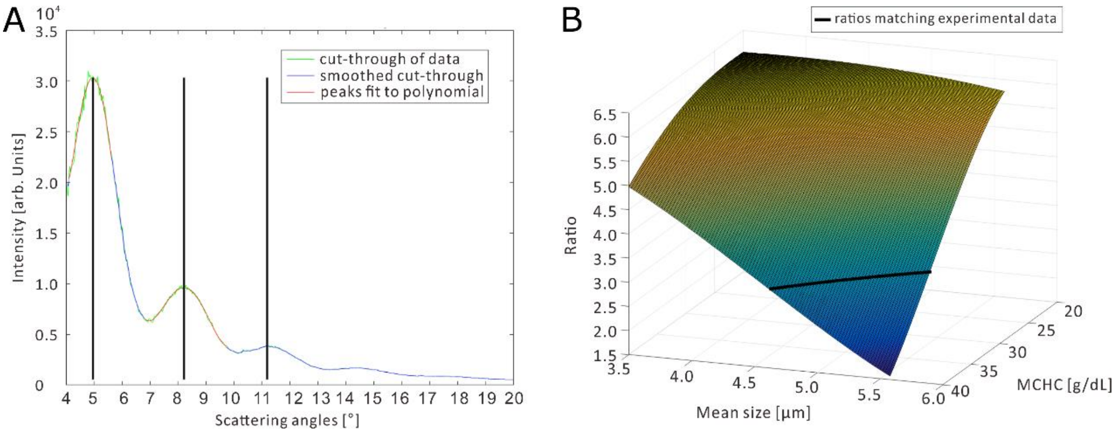

Figure S3 – Analysis of experimental data: (A) determining the location and peak heights of the first two peaks in the experimental curve; (B) comparing the ratio of the peak heights to the ratios (for single particles) of the entire theoretical database.

that the 655 laser was substantially weaker than the 405 nm laser (5mW vs. 20mW), and of course the scattering cross section of the particles decreases with increasing wavelength. These two factors combine to yield lower SNR for the 655nm laser compared to the 405 nm data (~16@655nm vs ~25@405nm). Further, the 655nm pattern is characterized by a wider fringe spacing with less prominent peaks and troughs. These combine to make it less robust than the 405nm data for the "peak-finding" step of the algorithm. However, the use of a second wavelength helps us in later stages of the algorithm when the sum squared error between experiment and theory is computed. This is because since the refractive index of the hemoglobin changes vs. wavelength, use of an additional wavelength provides independent information about the best combination of mean size and refractive index compared to one wavelength alone. 655 nm specifically was chosen only due to the availability of low-cost sources at this wavelength due to its use in CD players. In the future we plan to explore other cheaply available wavelengths (eg: 532 nm) to improve the robustness of the system. Following the successful fitting for the 405nm data, we then select every scattering curve in the theoretical database whose ratio (at 405nm) is similar to the experimental data, as shown in Figure S3B. This results in a vector of possible MCHC and MCV pairs. The theoretical data for identified MCHC-MCV pair is fit to the experimental data, with the best fit determining the MCHC and MCV. However, as we can see in the main text, the MCHC is the least accurately determined parameter. This is due to a sample dependent background on the data that corrupts the computation of the intensity ratio. We believe that this background may be due, in part, to scattering by platelets. As seen in Figure S4, scattering from a hypothetical anemic donor with an MCHC of 31 g/dL, MCV

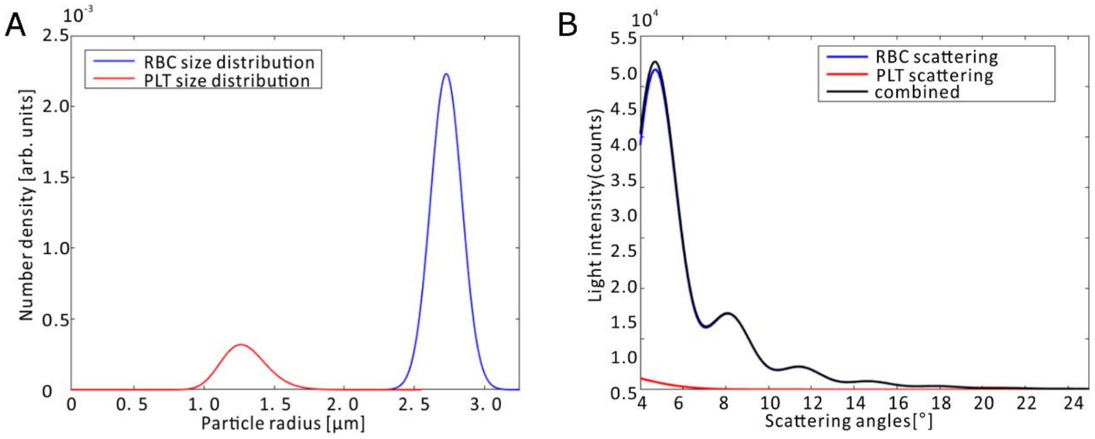

Figure S4 – potential influence of platelets on scattering curves. (A) Platelet versus red cell size distributions. (B) Platelet (red), Red cell (blue), and combined (black) scattering vs. angle for the size distributions shown in (A).

of 60 fL and RDW of 12% could be slightly influenced by scattering by platelets with a log-normal size distribution (shown in Figure S4A) where the platelet number is approximately 10% of the RBC number. The platelet scattering is significantly weaker than the red cell scattering due to their smaller size, number density, and refractive index contrast. However, at small angles they lead to a small but noticeable change in the height of the first peak (Figure S4B), potentially altering our MCHC estimation. However, the discrepancy between the two theoretical curves is still smaller than the discrepancy between the theoretical scattering and experimentally observed scattering shown in Figure 3E in the main text. Therefore, other background influences such as stray light due to dust or other factors must also be considered. Further work is needed to compensate for this issue.

Discrepancies notwithstanding, with determined MCV and MCHC, the last step is to determine the RDW value. Here we assume a Gaussian particle size distribution in form of:

$$\mathrm{G} = \exp\left(-\frac{(x-\mu)^2}{2\sigma^2}\right) \qquad \textbf{(Equation S4)}$$

where, x is the sphere size vector, μ is the sphere size, which is proportional to the MCV, found in the previous step and σ the standard deviation, related to the distribution width RDW. First, we need to create a model of scattering from multiple particles as the multiplication of a given size distribution with our theoretical size matrix:

$$\mathrm{M}(\lambda, \theta, \sigma) = \mathrm{G}(\mu, \sigma) \bullet T_{\lambda}(\mathrm{r}\ , c_{Hb}(\lambda), \theta\ ) + \mathrm{offset} \qquad \textbf{(Equation S5)}$$

Using the MCHC found in the previous step. The offset term is to try to compensate for the sample-dependent background described above. The experimental and the theoretical results are then compared with the goodness of fit and varying σ and the offset. In our case the goodness-of-fit is the Euclidean distance between the data and the model, and the distance between the logarithmic-scaled data and model:

$$E_{lin} = \sqrt{\sum_{i=1}^{n}[M_{si}(\lambda, \theta, \sigma) - I_{s\lambda i}(\mathrm{r}\ , c_{Hb}(\lambda), \theta)]^2} \qquad \textbf{(Equation S6)}$$

$$E_{log} = \sqrt{\sum_{i=1}^{n}[\log_{10} M_{si}(\lambda, \theta, \sigma) - \log_{10} I_{s\lambda i}(\mathrm{r}\ , c_{Hb}(\lambda), \theta)]^2} \qquad \textbf{(Equation S7)}$$

where E_{lin} is the Euclidean distance between the linear data, E_{log} is the Euclidean distance between the log-scale data, M_s the scaled experimental model and $I_{s\lambda}$ the experimental data. The final goodness of fit is F = E_{lin} + X*E_{log} (X represents the weighting factor, set empirically to 5×10^4 in our experiments). After this complex comparison between experiment and theory, MCV, MCHC, and RDW are determined. We can estimate the imprecision in our analysis method by

independently measuring the same blood sample multiple times, preparing each replicate separately and measuring in separate measurement chambers. Repeating the experiment 10 times, the imprecision was 1.25 fL in MCV, 1% in RDW, and 0.4 g/dL in MCHC.
